# Loss-of-function mutation of PCSK9 as a protective factor in the clinical expression of familial hypercholesterolemia: A case report

**DOI:** 10.1097/MD.0000000000021754

**Published:** 2020-08-21

**Authors:** Ane Bayona, Francisco Arrieta, Carmen Rodríguez-Jiménez, Francisco Cerrato, Sonia Rodríguez-Nóvoa, Milagros Fernández-Lucas, Diego Gómez-Coronado, Pedro Mata

**Affiliations:** a Department of Endocrinology and Nutrition, Ramón y Cajal University Hospital; b Ramón y Cajal Health Research Institute (IRYCIS); c CIBER of Pathophysiology of Obesity and Nutrition (CIBEROBN); d Department of Genetics of Metabolic Diseases, Institute of Medical & Molecular; Genetics (INGEMM), La Paz University Hospital; e Biochemistry-Research Department, Ramón y Cajal University Hospital; f Department of Nephrology, Ramón y Cajal University Hospital; g Fundación Hipercolesterolemia Familiar, Madrid, Spain.

**Keywords:** familial hypercholesterolemia, loss-of-function mutation, proprotein convertase subtilisin/kexin 9

## Abstract

**Rationale:**

Proprotein convertase subtilisin/kexin 9 or PCSK9 is a protein whose main function is to regulate the number of low-density lipoprotein receptors (LDLR) present on the cell surface. Loss-of-function mutations in *PCSK9* have been related to low LDL-cholesterol levels and a decrease in the risk of cardiovascular events.

**Patient concerns:**

We present the case of a 27-year-old woman, offspring of a patient with familial homozygous hypercholesterolemia, who presented with mild-moderate hypercholesterolemia.

**Diagnosis:**

Genetic analysis was performed by next generation sequencing using a customized panel of 198 genes. Sanger sequencing was used to confirm the presence of the variants of interest. The genetic analysis showed a pathogenic heterozygous mutation in *LDLR* [*exon 6:c.902A>G:*p(Asp301Gly)], as well as a loss-of-function heterozygous variant in *PCSK9* [*exon1:c.137 G>T:*p.(Arg46Leu)]. The genetic analysis of the index case's mother revealed compound heterozygosity for 2 different mutations in *LDLR* [*c.902A>G:*p.(Asp301Gly); *c.1646G>T:*p.(Gly549Val)] in exon 6 and in exon 11, respectively, and the same loss-of-function variant in *PCSK9* that had been found in her daughter [(*PCSK9:exon1:c.137G>T:*p.(Arg46Leu)]. The maternal grandfather of the index case presented the same genetic variants as his granddaughter.

**Interventions:**

The index case did not receive any specific treatment for hypercholesterolemia. The loss-of-function variant in *PCSK9* protected her from higher LDL-cholesterol levels, provided she kept partial activity of the LDLR. In her mother, instead, a *PCSK9* inhibitor was tried but failed to achieve lipid control. The reason for this may be the complete absence in LDL receptor activity. LDL apheresis was started afterwards, resulting in adequate lipid level control.

**Outcomes:**

To the date, the index case has achieved to maintain adequate total and LDL-cholesterol levels without any other intervention. She has had no known cardiovascular complication.

**Lessons:**

Loss-of-function mutations in *PCSK9* could protect from developing more severe forms of hypercholesterolemia. The finding of these mutations (*LDLR-PCSK9*) in three consecutive generations could imply an adaptive mechanism against the development of hypercholesterolemia.

## 1 Introduction

Proprotein convertase subtilisin/kexin 9 or PCSK9 is a 72-kDa molecule containing 692 amino acids whose main function is binding to specific cell surface receptors to escort them toward intracellular acidic endosome/lysosome degradation compartments. PCSK9 is secreted by the liver and has a main role in cholesterol metabolism, acting as an endogenous inhibitor of low-density lipoprotein receptor (LDLR). Thus, it lowers the hepatic uptake of LDL-cholesterol (LDL-c) by increasing the endosomal and lysosomal degradation of LDLR, therefore decreasing their number on the cell surface.^[1]^

Gain-of-function mutations in *PCSK9* have been associated to hypercholesterolemia and to an increase in cardiovascular risk.^[2]^ In contrast, loss-of-function mutations in *PCSK9* result in low LDL-c levels and seem to decrease the risk of coronary heart disease, without having any known negative impact on human health.^[3]^ Consistently with the effect of loss-of-function mutations, several clinical trials have demonstrated that PCSK9 inhibition potently reduces serum LDL-c concentrations.^[4]^ PCSK9 inhibitors have recently emerged as highly effective hypolipidemic drugs, given alone or in combination with statins or a statin plus ezetimibe.

Familial hypercholesterolemia (FH; MIM# 143890), a disease most often inherited in an autosomal dominant manner, comprises a series of anomalies in LDL metabolism that result in hypercholesterolemia of variable severity due to a genetic dosing effect. This way, patients with homozygous FH (HoFH) present with higher levels of total and LDL-c than patients with heterozygous forms. Hypercholesterolemia is caused by disruption of genes which are critical to LDL catabolism, mainly the *LDLR* gene (MIM# 606945), the *APOB* gene (MIM# 107730) and the already mentioned gain-of-function mutations in *PCSK9* (MIM# 607786), the latter being responsible for a small percentage (<2%) of the cases of FH overall. Moreover, mutations in *LDLRAP1* (MIM# 605747) cause an autosomal recessive form of FH (ARH), which also has low prevalence.^[5]^

## 2 Methods

Here, we present the case of a 27-year-old woman, offspring of a patient with familial homozygous hypercholesterolemia, who presented with mild-moderate hypercholesterolemia. Genetic analysis was performed by next generation sequencing (NGS). Local ethics committee approved this study and the patients gave written consent for the publication of the results.

To perform the genetic analysis, the genomic DNA (gDNA) was extracted from Ethylenediaminetetraacetic acid (EDTA) treated whole blood samples using Chemagen (Chemagic DNA extraction special, Perkin Elmer Inc, Baesweiler, Germany). DNA quantification was performed using a NanoDrop ND-1000 Spectrophotometer. Genetic analysis was performed by NGS using a customized panel of 198 genes. Library preparation and exome enrichment steps were performed according to manufacturer's workflow (Nimblegen, Roche, Basel, Switzerland) and it was sequenced using NextSeq system Sequencing (Illumina, San Diego, California, US). A subset of genes was chosen for analysis of hypercholesterolemia: *LDLR*, *APOB*, *PCSK9*, and *LDLRAP1*. NGS data were suitable for analysis after passing the quality parameters established in our laboratory, which consisted in a number of reads of more than 30× in the 99% of the target bases. Sanger sequencing was used to confirm the presence of the genetic variants found. The multiplex ligation-dependent probe amplification (Salsa P062-D2 kit; MRC-Holland, Amsterdam, The Netherlands) was used for the detection of large rearrangements in *LDLR* according to manufacturer's instructions.

Furthermore, the measurement of LDLR activity was carried out by incubating the patient's peripheral blood lymphocytes with different concentrations of human DiI-labeled LDL at 4°C (binding) or 37°C (uptake) and subsequent analysis by flow cytometry as previously described.^[5]^

## 3 Case report

We describe the case of a 27-year-old Caucasian woman, offspring of a woman with HoFH, who presented with mild-moderate hypercholesterolemia (LDL-c levels of 167 mg/dL [4.3 mmol/L] and total cholesterol of 237 mg/dL [6.1 mmol/L]). The calculated *Dutch Lipid Clinic Network* score was of 4 (possible diagnosis of FH). Otherwise, she was a healthy woman without any relevant medical or surgical history. She was asymptomatic, presented no relevant findings in the physical examination and she was not taking any cholesterol lowering medication. The presence of mild to moderate hypercholesterolemia in the presented case, given the family history of HoFH, motivated further study by genetic analysis.

The mother of the index case, a 54-year-old woman, had been followed up for HoFH. She had been diagnosed at the age of 7, after developing elbow xantomata. She fulfilled the criteria for clinical diagnosis of HoFH.^[6]^ The patient has been treated with LDL apheresis every 15 days since she was 29 years old. The pre-apheresis LDL cholesterol levels were of 351 mg/dL (9.1 mmol/L) and total cholesterol levels of 415 mg/dL (10.7 mmol/L). In 2016, the PCSK9 inhibitor evolocumab (*Repatha*) was introduced, resulting in a minimal improvement in LDL-c levels. She had a history of aortic valve disease with severe stenosis that needed valve replacement surgery when she was 38 years old (metallic aortic ATS18). There was no history of any other cardiovascular events.

**Figure 1:**
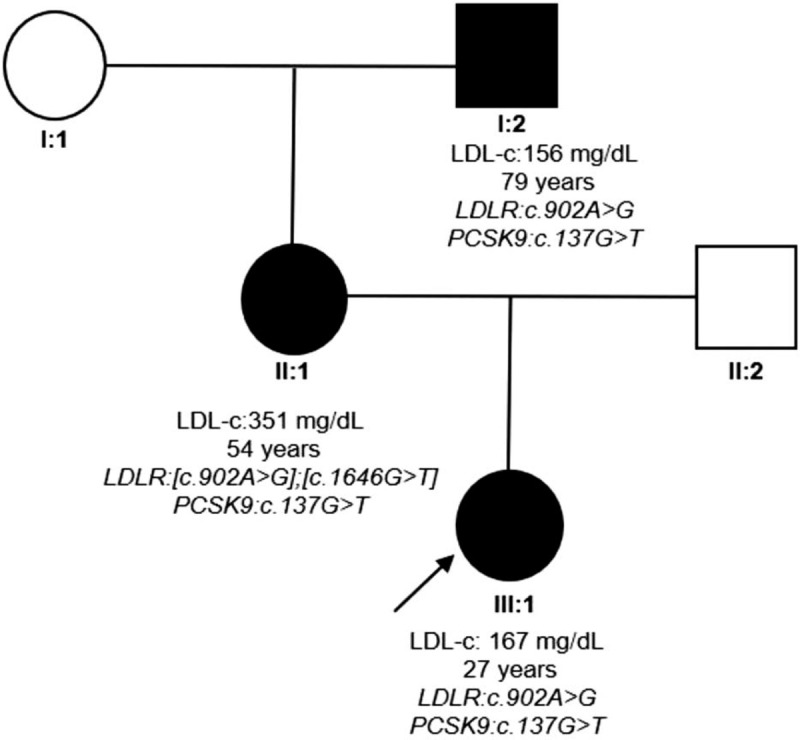
Pedigree of the family. The index case is indicated with an arrow; circle and square symbols represent women and men, respectively; shadow filled symbols indicate the affected members with familial hypercholesterolemia. Line 1 below symbols corresponds to the individual identification, line 2 indicates LDL-c (levels without lipid-lowering therapy), line 3 indicates age, and lines 4 and 5 indicate the genotype for *LDLR* and *PCSK9*, respectively. *LDLR* = low-density lipoprotein receptor, *PCSK9* = proprotein convertase subtilisin/kexin 9.

The maternal grandfather (79 years old) of the index case also presented with mild hypercholesterolemia (LDL-c levels of 156 mg/dL [4.034 mmol/L] and total cholesterol levels of 235 mg/dL [6.1 mmol/L]). He had a history of stroke at an age older than 65 years. The Figure 1 shows a pedigree of the family with the affected members represented in black. The genetic analysis of the index case found a loss-of-function variant in *PCSK9 c.137G>T:*p.(Arg46Leu), besides a pathogenic mutation in *LDLR c.902A>G:*p.(Asp301Gly), both in heterozygosity. Her mother, aside from showing the presence of 2 different mutations in *LDLR* [*c.902A>G;*p.(Asp301Gly)]; [*c.1646G>T:*p.(Gly549Val)] in exons 6 and 11 respectively, was also a carrier of the same heterozygous mutation in *PCSK9* found in her offspring. The maternal grandfather's genetics revealed the same mutations found in the index case (Table 1).

**Table 1:**
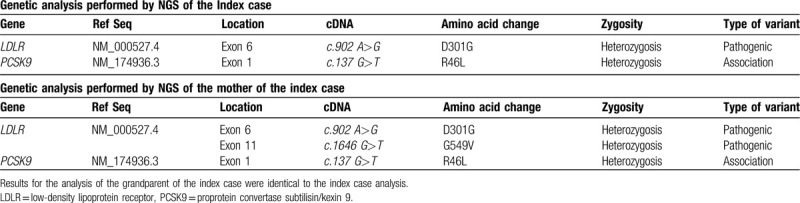
Results from the genetic analysis performed by massive genome sequencing of the index case and mother of the index case.

Regarding the LDLR activity analysis, the mother of the index case showed undetectable LDL binding and uptake, consistent with her homozygous LDLR deficiency. In the index case, the binding activity was 64% and the uptake activity was 81% vs the control, indicating a partial deficiency of LDLR activity. An analysis of the lymphocytes from a healthy normolipidemic cousin of the index case's mother showed a binding activity of 127% and an uptake activity of 141% vs the control.

## 4 Discussion

We report the case of a woman with mild to moderate hypercholesterolemia despite being the offspring of a patient with HoFH. The genetic analysis showed the presence of a loss-of-function mutation in *PCSK9 c.137G>T:*p.(Arg46Leu), in addition to a pathologic mutation in *LDLR c.902A>G:*p.(Asp301Gly), both in heterozygosity. To our knowledge, this is the first report of the simultaneous inheritance of a pathologic mutation in *LDLR* and a loss-of-function variant in *PCSK9* in family members from three consecutive generations.

The *LDLR c.902A>G:*p(Asp301Gly) variant was first reported in a Greek FH patients by Mavroidis et al in 1997^[7]^ and subsequently it was found in Dutch FH patients.^[8]^ In 2015, the *LDLR c.902A>G*:p(Asp301Gly) was classified by Etxebarria et al as a pathogenic, class 3 mutation, the functional study being carried out in transfected CHO-*ldlA7* cells, which lack functional endogenous LDLR.^[9]^ The analysis of LDLR activity performed by these authors revealed a 100% surface expression of the receptor compared to wild type, but LDL binding was decreased by at least 50% and the uptake was <40% compared to the control. As expected, in the case we present, a heterozygote for *LDLR c.902A>G*:p(Asp301Gly), her lymphocytes showed LDLR binding activity and uptake activity higher than in cells carrying only this *LDLR* mutation.

In 2001, another Spanish group, Chaves et al, conducted a study aiming to ascertain if the molecular analysis of *LDLR* helped in predicting the response to simvastatin in heterozygous FH individuals.^[10]^ They provided total cholesterol and LDL-c levels for a group of subjects with FH and different *LDLR* mutations, including class 3 defective mutations such as *LDLR c.902A>G:*p (Asp301Gly). They reported a mean total cholesterol of 368 mg/dL (9.51 mmol/L) and LDL-c levels of 288 mg/dL (7.47 mmol/L) in these subjects before starting on simvastatin, much higher than total cholesterol and LDL-c levels that presented our case (237 mg/dL [6.1 mmol/L] and 167 mg/dL [4.3 mmol/L], respectively). This is attributable to the cholesterol lowering effect exerted by the PCSK9 variant that coexists in this subject.

Isolated loss-of-function mutations in *PCSK9* have been associated to a mild to moderate decrease in LDL-c levels (between 15% and 28%) and they may confer significant lifelong cardiovascular protection.^[3]^ Compound heterozygous cases with 2 different inactivating mutations in *PCSK9* have been reported. In these patients, secreted PCSK9 is absent; it seems, however, that PCSK9 secretion is not essential for the normal development and performance of the individual.^[3]^

The p.(Arg46Leu) variant, resulting from *c.137G>T* in *PCSK9*, was found in the index case, her mother and the maternal grandfather. This is a loss-of-function variant frequently reported in Caucasians (>2%) and seems to lower LDL-c levels by 11% to 16%, the risk of coronary heart disease more than expected for the LDL-c reduction, and also the risk of aortic valve disease.^[3]^

There are 2 remarkable aspects in the case presented. On the one hand, the index case, presented with mild-moderate hypercholesterolemia despite being the offspring of a patient with HoFH, which led to the genetic analysis. Both the index case and her maternal grandfather had 2 heterozygous mutations with opposite effects: a pathologic mutation in *LDLR c.902A>G:*p.(Asp301Gly) and a loss-of-function mutation in *PCSK9 c.137G>T:*p.(Arg46Leu). None of the 2 subjects had any history of cardiovascular disease at a premature age. The presence of the *PCSK9* mutation may have protected them from the development of a severe form of hypercholesterolemia, resulting in a mild to moderate hypercholesterolemia despite the presence of a pathologic mutation in *LDLR*.

On the other hand, the study of LDLR activity showed total deficiency of this activity in the case's mother, while there was only a partial deficiency of LDLR activity in the index case. Total deficiency of LDLR activity may explain the limited effect of pharmacologic inhibition of PCSK9 in this patient. This fact agrees with the previously reported lower efficacy of PCSK9 inhibitors in HoFH patients with negative LDLR activity (<2%) than in homozygotes with higher residual LDLR activity. Also, the loss-of-function mutation in PCSK9 may neither confer the cardiovascular protection nor have the cholesterol lowering effect in this patient compared to the index case, who keeps significant LDLR activity.

The main limitation of this study is the small kindred. Unfortunately, we did not have the possibility to assess any other family member due to the lack of other relatives in the family.

## 5 Conclusion

In summary, we present the case of an otherwise healthy woman with mild to moderate hypercholesterolemia, despite being the offspring of a patient with HoFH. The genetic analysis showed the presence of a loss-of-function mutation in *PCSK9*, that is, *c.137G>T:*p.(Arg46Leu), in addition to a pathologic mutation in *LDLR c.902A>G:*p.(Asp301Gly), both in heterozygosity. Furthermore, the presence of a loss-of-function mutation in *PCSK9* seems to have a protective effect against the development of severe forms of hypercholesterolemia that the coincident mutation in *LDLR* could have caused, as seen in the index case and her grandfather.

## Author contributions

AB, MLF and FA designed the study and participated in the selection of patients and data acquisition. AB, FA and PM participated in writing the manuscript. CRJ and SRN performed the genetic analysis. DGC and FC performed the analysis of LDLR activity. All authors have revised the article critically and approved the final version for submission.
